# Enhancement and Function of the Piezoelectric Effect in Polymer Nanofibers

**DOI:** 10.1021/accountsmr.2c00073

**Published:** 2022-08-15

**Authors:** Luana Persano, Sujoy Kumar Ghosh, Dario Pisignano

**Affiliations:** † NEST, Istituto Nanoscienze-CNR and Scuola Normale Superiore, Piazza San Silvestro 12, I-56127 Pisa, Italy; ‡ Dipartimento di Fisica, Università di Pisa, Largo B. Pontecorvo 3, I-56127 Pisa, Italy

## Abstract

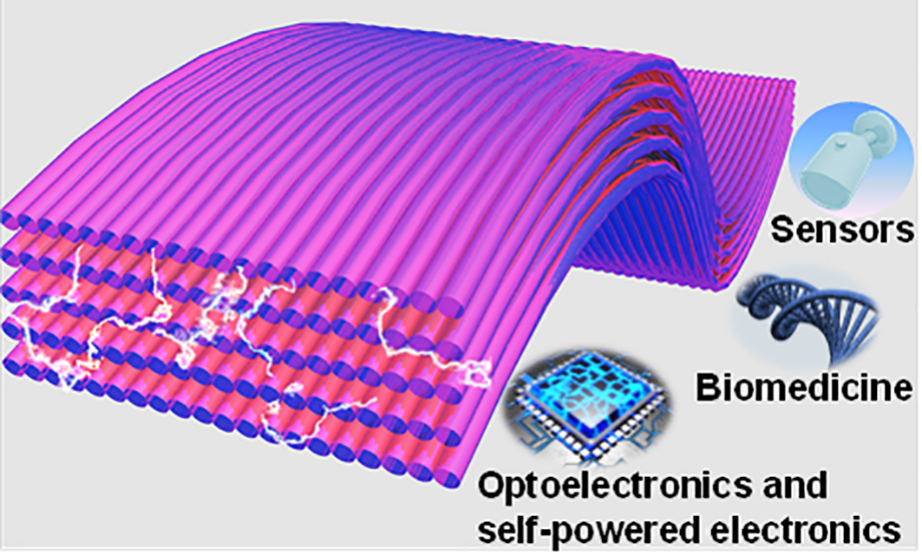

The realization of intelligent, self-powered components and devices exploiting the piezoelectric effect at large scale might greatly contribute to improve our efficiency in using resources, albeit a profound redesign of the materials and architectures used in current electronic systems would be necessary. Piezoelectricity is a property of certain materials to generate an electrical bias in response to a mechanical deformation. This effect enables energy to be harvested from strain and vibration modes, and to sustain the power of actuators, transducers, and sensors in integrated networks, such as those necessary for the Internet of Thing. Polymers, combining structural flexibility with lightweight construction and ease of processing, have been largely used in this framework. In particular, the poly(vinylidene fluoride) [PVDF, (CH_2_CF_2_)_*n*_] and its copolymers exhibit strong piezoelectric response, are biocompatibile, can endure large strains and can be easily shaped in the form of nanomaterials. Confined geometries, improving crystal orientation and enhancing piezoelectricity enable the fabrication of piezoelectric nanogenerators, which satisfy many important technological requirements, such as conformability, cheap fabrication, self-powering, and operation with low-frequency mechanical inputs (Hz scale). This account reports on piezoelectric polymer nanofibers made by electrospinning. This technique enables the formation of high-aspect-ratio filaments, such as nanowires and nanofibers, through the application of high electric fields (i.e., on the order of hundreds of kV/m) and stretching forces to a polymeric solution. The solution might be charged with functional, organic or inorganic, fillers or dopants. The solution is then fed at a controlled flow rate through a metallic spinneret or forms a bath volume, from which nanofibers are delivered. Fibers are then collected onto metallic surfaces, and upon a change of the collecting geometry, they can form nonwovens, controlled arrays, or isolated features. Nanofibers show unique features, which include their versatility in terms of achievable chemical composition and chemico-physical properties. In addition, electrospinning can be up-scaled for industrial production. Insight into the energy generation mechanism and how the interaction among fibers can be used to enhance the piezoelectric performance are given in this paper, followed by an overview of fiber networks as the active layer in different device geometries for sensing, monitoring, and signal recognition. The use of biodegradable polymers, both natural and synthetic, as critically important building blocks of the roadmap for next-generation piezoelectric devices, is also discussed, with some representative examples. In particular, biodegradable materials have been utilized for applications related to life science, such as the realization of active scaffolds and of electronic devices to be placed in intimate contact with living tissues and organs. Overall, these materials show many relevant properties that can be of very high importance for building next-generation, sustainable energy harvesting, self-rechargeable devices and electronic components, for use in several different fields.

## 1 Introduction

The progress toward sustainable development has been frequently limited, partly because of other planetary emergencies including pandemics, making the delivery on the 2030 Agenda and the 2015 Paris Agreement on Climate Change very hard to accomplish.^1^ The unsustainable use of natural resources has been essentially driven by a continuous economic growth and industrial development. The industrial sector consumes about 54% of the world’s total delivered energy,^2^ followed by transportation (with road transportation generating 75% of the carbon dioxide emission of the entire sector).^3^ However, recent crises have highlighted more than ever how such a trend not only deteriorates our natural environment but might also ultimately affect human health, inequalities, and migrations at global scale. Technological development also evidences that common laws and policies as well as global scientific collaborations for the achievement of large-scale goals are affordable, and present crises can offer unique opportunities to remodel our whole system of industrial progress and energy management, targeting a definitive reduction of carbon emissions and improvement of our efficiency in using resources. In the framework of the 17 global goals for a sustainable future included in the 2030 Agenda, goal 7 seeks to “Ensure access to affordable, reliable, sustainable and modern energy for all”. The use of renewable energy sources is growing by exploiting mainly solar, wind, hydroelectric, and geothermic energies. The realization of intelligent, self-powered components, devices and networks at large scale, possibly embedding a large variety of sensing, monitoring, actuation and communication functions, might greatly contribute to these global objectives, but it would need a profound redesign of the materials and architectures used in our electronic systems. Piezoelectricity, which is a property of certain materials to generate electrical charges in response to a mechanical deformation, can generate tens of mW per m^2^ by exploiting strain and vibration modes, and hence it can sustain the power of actuators, transducers and sensors necessary to build integrated networks. In the forthcoming Internet of Things, this capability can be additionally exploited for applications such as environmental monitoring,^4^ robotics,^5^ smart homes,^6^ and healthcare.^7^

Piezoelectricity is observed in materials whose crystalline state has no center of symmetry (so-called noncentrosymmetric), and it is largely related to the presence of electric dipoles.^8^ When dipoles are mutually aligned within certain spatial regions (Weiss domains), they produce a neat macroscopic polarization vector, **P**. Such alignment can occur spontaneously, or for some materials (*ferroelectrics*), it can be forced by an externally applied, intense electric field (*poling* procedure), or by mechanical drawing. Following a mechanical stress (σ), the intensity and/or the direction of **P** may then vary as well as the strain, ε, induced in the material. The material electro-mechanical behavior is then described in terms of piezoelectric coefficients, *d*_*ij*_ = *∂D*_*i*_/*∂σ*_*j*_, for *i* = 1–3 and *j* = 1–6, where *D*_*i*_ are the components of the electric displacement, the direction numbered with 3 is, by convention, identified with the direction of pristine polarization (resulting from the fabrication or poling process). The *i* subscript in *d*_*ij*_ indicates the direction (i.e., *x*, *y*, or *z*) of the electric displacement resulting in the piezoelectric material, namely, the direction along which a voltage bias is generated, whereas the *j* subscript indicates the direction of applied stress (for *j* = 1–3), also specifying if shear-stress is involved (for *j* = 4–6). Shear piezoelectric response, observed in several uniaxially oriented systems,^9−11^ can be very useful for the versatile design of a variety of flexible devices.

Inorganic materials such as lead zirconate titanate have been commonly used because of the excellent piezoelectric properties^12^ and high Curie temperature (>200 °C). However, the well-established neurotoxic effects of lead, especially for the developing brain, lead to strong issues, and regulations have been gradually introduced from the European Commission and the United States environment protection agencies to cut down its use in many industrial products, such as paints, food cans, and water pipes.^13,14^ Nowadays, the development of lead-free piezoelectric materials is highly desired. Polymers, which combine structural flexibility with lightweight constructions and ease of processing, are important candidates in this respect. They have been largely used for energy applications. For instance, polymers exhibiting piezoelectric properties include PVDF, nylon, poly(l-lactic acid) (PLLA), poly(lactide-*co*-glycolide), and natural biomaterials such as silk, chitin, gelatin and cellulose. Examples of technologies based on piezoelectric polymers are hydrophones,^15^ sensors,^16,17^ and actuators.^18^

PVDF and its copolymers exhibit strong piezoelectric response,^19,20^ are biocompatibile, and can endure large strains.^3^ PVDF-based materials have a semicrystalline structure, where microscopic crystals are randomly distributed within an amorphous phase. Different regions (crystalline, amorphous, and amorphous–crystalline interfaces) coexist in the material, and various models have been proposed to explain the origin of piezoelectricity in PVDF by considering the contribution of both electrical dipoles and the deformation of the amorphous regions.^21^ Four different crystalline phases have been studied, named α (TGTG conformation, where T = trans and G = gauche), β (all T-conformation), δ, and γ. In addition, PVDF features a negative piezoelectric coefficient [*d*_33_]. Negative longitudinal piezoelectric coefficients indicate a contraction of the material upon the application of an electric field pointing along the polarization direction), whose behavior is the object of experimental and theoretical investigation.^21−23^ The recent demonstration of morphotropic phase boundary in poly(vinylidenefluoride-*co*-trifluoroethylene) P(VDF-TrFE) suggests a crystalline origin of the negative longitudinal piezoelectric coefficient and opens interesting perspectives for the improvement of the piezoelectric response by polymer chain engineering.^21^

Piezoelectric polymers can be easily shaped in the form of nanomaterials. The use of nanomaterials for building energy harvesting devices and electronic components relies on the flexibility of the design, the miniaturization, and the frequently enhanced output performance. Indeed, confined geometries improve crystal orientation^24^ and they can enhance piezoelectricity leading to the formation of the preferred crystalline phase.^25^ The use of piezoelectric nanogenerators, converting mechanical energy into electricity, have the potential to push forward the development of integrated and interconnected networks of sensors and actuators, because they satisfy requirements such as conformability, cheap fabrication technology, self-powering, and accurate operation with low-frequency mechanical inputs (Hz scale).^26^

In the broad arena of nanomaterials, polymer nanofibers are very thin and long filaments, having lateral size potentially down to the scale of tens of nanometers, and length orders of magnitude larger than their diameter. They can be assembled to form nonwovens or be used as single elements. Among the technologies introduced to form these materials, electrospinning, which uses high electric fields and stretching forces to form long and thin^27^ fibers, has unique features, which include the operational simplicity and chemical versatility. In addition, electrospinning can be up-scaled for industrial production.^28^ This Account reports on polymer piezoelectric nanofibers made by electrospinning, with insight into the energy generation mechanism and how the mutual interaction among fibers can be used to enhance the piezoelectric performance. We provide an overview of fiber networks as the piezoelectric active layer in different device geometries for sensing, monitoring, and signal recognition. The use of biodegradable polymers, both natural and synthetic, as a critically important building block of the roadmap for next-generation piezoelectric devices is also discussed, with some representative examples.

## 2 Electrospinning Technology for Piezoelectric Polymers

The electrospinning process is based on the use of (i) a highly concentrated polymer solution (i.e., 5%–30% in polymer weight with respect to solvent), delivered in a continuous way through a free liquid surface or a spinneret, and (ii) the application of a high electric field (typically 10^5^–10^6^ V/m) between the solution and a metal surface, named collector. When metal needles, or capillaries, are used as the spinneret, their internal diameter is generally on the order of hundreds of micrometers. High polymer concentrations are needed to lead to molecular entanglements in the solution, thus providing the fluid with significant viscoelastic properties. A jet is formed when the force because of the external electric field overcomes surface tension and viscoelastic forces in the fluid. Once formed, the jet proceeds toward the collector with speed in the range 10–100 cm/s and large acceleration (10^4^–10^5^ cm/s^2^), which leads to very high strain rates, exceeding the reciprocal relaxation time of the polymer solution, and possibly causing stretching of the polymer chains thus producing some degree of orientational anisotropy of molecules within the produced fibers.^29^ During the flight of the jet from the spinneret to the collector, the solvent evaporates and the jet cross-sectional size is reduced by some orders of magnitude. Nanofibers are then collected on the metal surface and, according to the collector geometry, they can have specific orientations and configurations. Overall, the electrospinning process is governed by several parameters which can be grouped into three main classes: (I) variables related to solution properties (e.g., concentration, viscosity, electrical conductivity), (II) variables related to the used setup (e.g., applied voltage, solution flow rate, spinneret to collector distance), and (III) ambient variables (temperature, humidity and pressure).^29,30^ Depending on such parameters, electrospun fibers might exhibit very different cross-sectional diameters and surface properties.

PVDF and its copolymers such as P(VDF–TrFE) have been widely used for the realization of piezoelectric devices based on electrospun nanofibers.^31,32^ The combination of high electric fields and large stretching forces in electrospinning induce significant anisotropy in these polymers, intrinsically causing some local poling and, by consequence, superior piezoelectric performance in the resulting fibers, without need of additional electrical poling or drawing. In fact, piezoelectric properties are deeply affected by the fiber properties. Finer fibers (average diameter below 300 nm) generally show enhanced electrical outputs regardless of the electrospinning parameters used (variables of class I and II mentioned above)^33^ whereas environments with higher humidity (60% vs 30%) provide higher β-phase content and larger piezoelectric coefficients. An important role on the surface chemistry appears to be also played by the polarity of the voltage applied to the spinneret, which reduces the number of fluorine groups at the surface, yielding to enhanced piezoelectric performances as recently found by an international collaboration with the Kar-Narayan and Stachewicz groups.^30^ A more extensive discussion on electrospinning parameters that affect the piezoelectric response is reported in ref (34).

**Figure 1:**
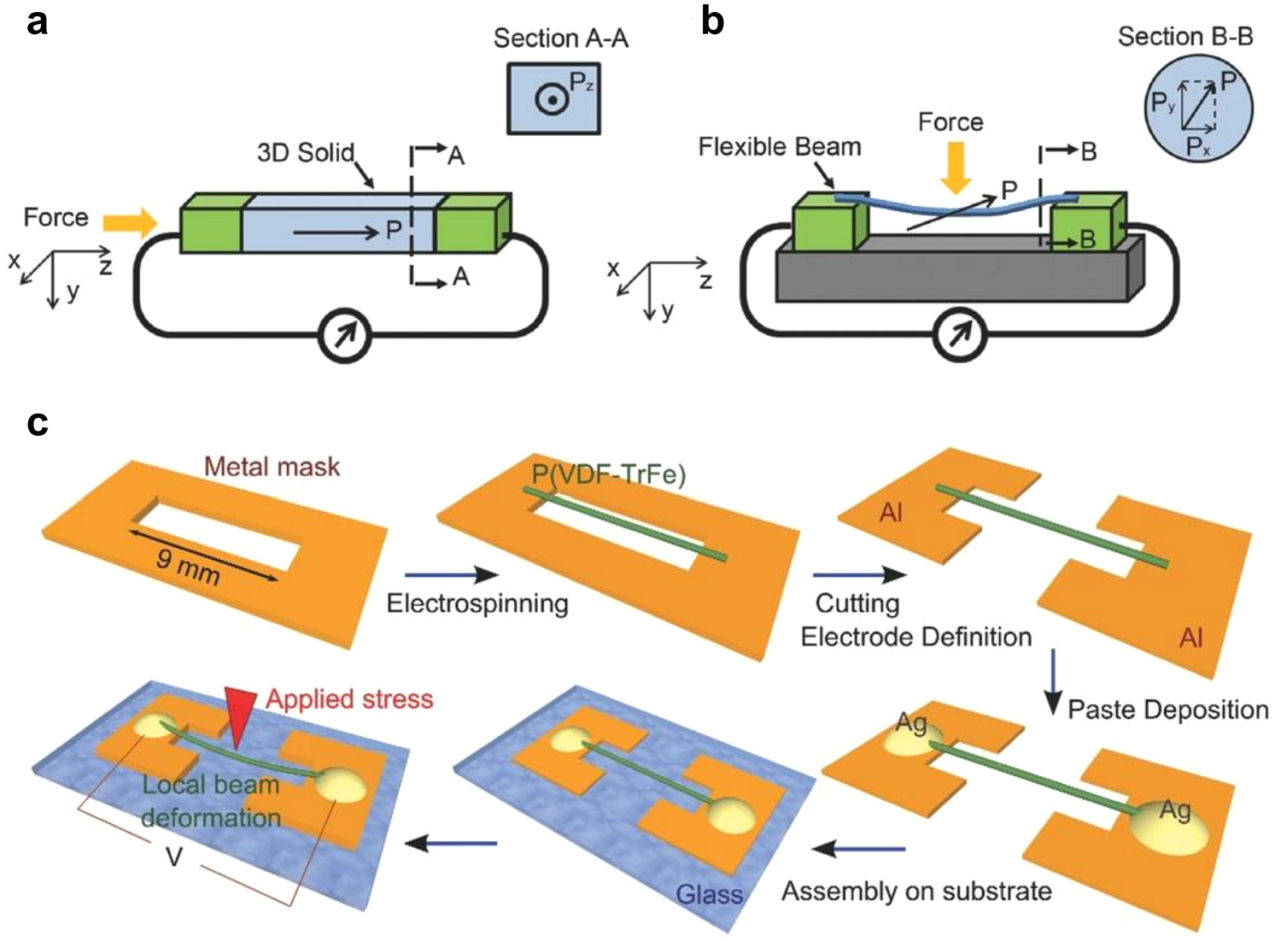
Normal vs shear piezoelectricity in nanofibers. Schematics (a) of normal piezoelectricity in a 3D crystalline solid, and (b) of shear piezoelectricity in nanofibers. (c) Schematics of the processing steps for the fabrication of piezoelectric devices based on a single suspended nanofiber. Reproduced with permission from ref (10). Copyright 2016 John Wiley and Sons, Inc.

In our studies, we have mainly worked with PVDF-TrFE (ratio of copolymer: 0.73:0.27), which generally exhibits high piezoelectric coefficients^20^ and a stable β-phase at room temperature. An electrical response can originate even along directions perpendicular to the applied stress. In addition, by considering the varied degree of alignment of the polymer chains, the concept of uniaxial piezoelectricity cannot be applied, and more complex transverse contributions are to be taken into account to properly define polarization directions. Studying the biaxial shear activity in P(VDF-TrFE) leads to highlighting two net components of the electronic polarization in the plane perpendicular to the chains of macromolecules (Figure 1a,b).^10^ The microscopic shear stress can be exploited in single P(VDF-TrFE) fibers with average diameters of 400–600 nm and high length/diameter ratios (up to 5 × 10^5^), in suspended device configurations on either stiff or flexible substrates (Figure 1c). A strain perpendicular to the longitudinal axis of the fiber, producing a localized bending depth up to ∼100 nm, can be applied by the use of a nanoindenter. A 40 μV peak voltage is generated in response to the applied strain. Ab initio calculations, carried out by our collaborators Catellani and Calzolari,^10^ allowed the piezoelectric tensors to be assessed, together with the Born effective charges (Z* = −Ω∂**P**/∂**r**, where Ω is the unit cell volume and **r** indicates the coordinate of an atomic displacement) because of displacement-induced polarization changes. This analysis highlights that multiple shear components are nonzero in P(VDF-TrFE). By comparison with bare PVDF, the inclusion of TrFE units is found to strongly increase the repulsion among fluorine atoms and to lead to the establishment of a transversal component of the **P** vector, i.e., to a biaxial polarization character. An analytic electromechanical model, developed by the Huang group at Northwestern University through the ab initio calculated piezoelectric coefficients,^10^ confirms the substantial contribution that the shear stress makes to the output voltage and indicates that the shear piezoelectric voltage depends strongly on the position of the applied strain with respect to the fixed edges of the fibers. Overall, these results make piezoelectric polymer nanofibers highly interesting for detecting eccentric loads and for nanoscale position sensing.

At the nanoscale, the effect of shear strain on single piezoelectric fibers can be measured by lateral piezoresponse force microscopy (LPFM), where in-plane displacement arises from the torsional mechanical forces between the PFM cantilever tip and the sample. In electrospun PVDF nanofibers, LPFM results support the presence of two transverse polarization components,^35^ whereas in single fibers of P(VDF-TrFE) made by melt-electrowriting (a solvent-free additive manufacturing technique), it additionally suggests different dynamics of crystallization and solidification along the fiber backbone. Electro-writing of highly viscous P(VDF-TrFE) melts was achieved by means of collectors at >120 °C, with slow collector speeds (<100 mm/min), and LPFM was applied to the so-obtained fibers in the framework of a collaboration with the Luxenhofer and Dalton groups.^36^ Other teams have demonstrated the relevant contribution of transverse coefficients to piezoelectricity in different relevant materials, such as thin films of halide perovskytes^37^ and organic–inorganic hybrid perovskite nanorods.^38^

## 3 Intercoupling Effects Enhance Piezoelectric and Other Functional Properties of Nanofibers

**Figure 2:**
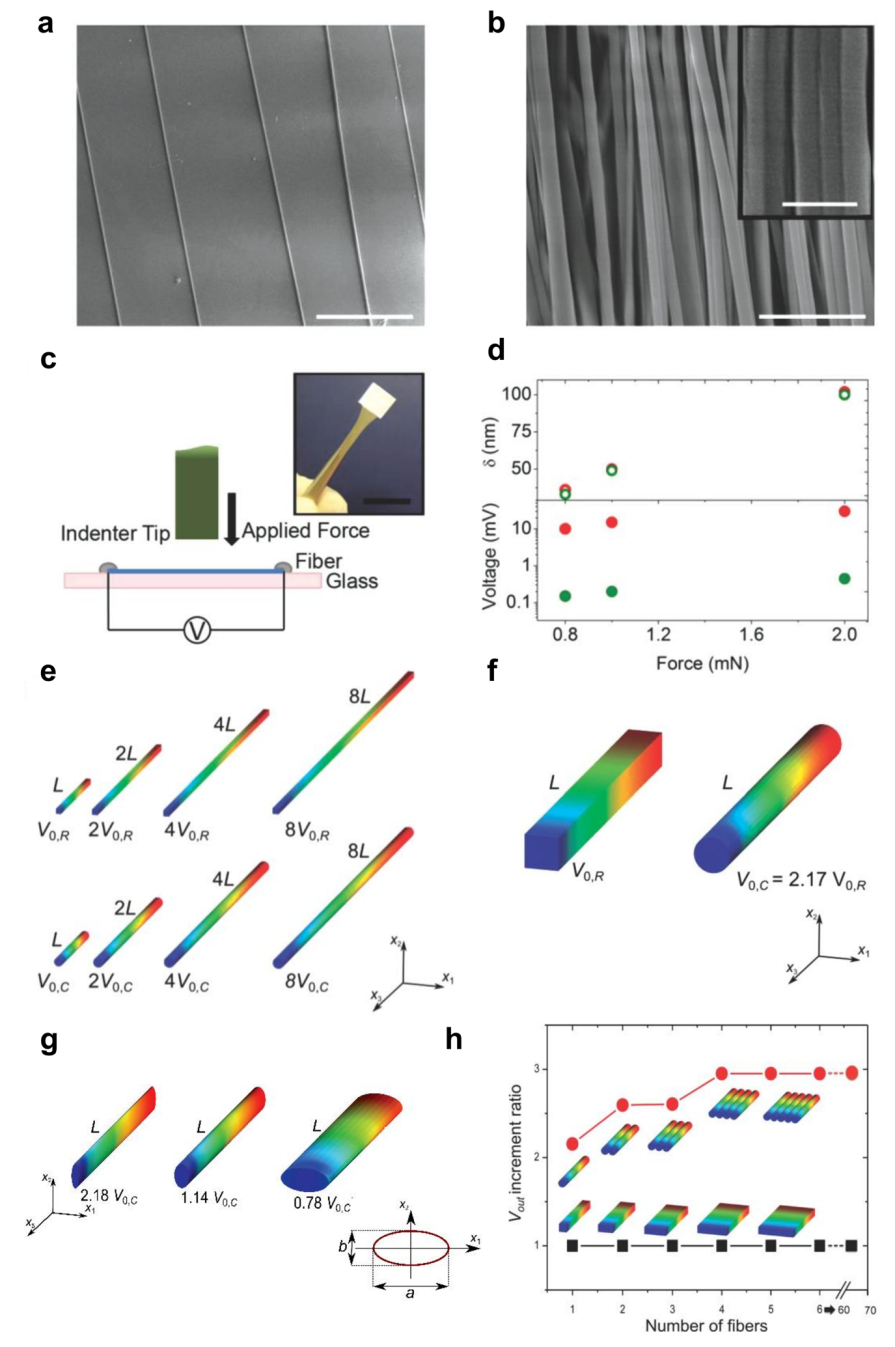
Cooperativity in the enhanced piezoelectric response of arrays of nanofibers. SEM images (a) of an array of isolated fibers (scale bar, 20 μm) and (b) of a dense array of fibers in mutual contact (scale bar, 3 μm). Inset in (b): high-magnification view (scale bar, 1 μm). (c) Experimental setup for force–indentation measurements. (d) Measured displacement, δ, and voltage response (green dots, array of isolated fiber; red dots, dense array of fibers). (e) Numerical simulation of the voltage distribution on the surface of nanofibers depending on the fiber length (*L*). *R* and *C* indicate differently shaped cross sections of the fiber: rectangular and circular, respectively. Red (blue) corresponds to high (low) voltage values. (f, g) Comparison of the voltage distribution on the surface of nanofibers having different shapes (f) and elliptical sections (g). (h) Dependence of the voltage distribution for horizontally (in-plane) stacked fibers. Adapted with permission from ref (39). Copyright 2014 John Wiley and Sons, Inc.

Additional work aimed to investigate the piezoelectric behavior of electrospun fibers in the form of an array, made of either isolated (not in contact) or densely packed (10^7^ fibers/mm^2^) fibers (Figure 2a,b).^39^ These studies were performed by us in the framework of a collaboration with Dagdeviren and De Lorenzis group^39^ carrying our extensive finite element multiphysics simulations. Normal forces in the millinewton range can be easily delivered to the fibers by the use of a tribo-indenter (Figure 2c) equipped with a flat ended cylinder sapphire tip (1 mm diameter). Measured displacements are in the interval 30–100 nm, with no significant difference between isolated fibers and arrays under the same applied force (Figure 2d). On the contrary, the voltage output generated by the array is up to 2 orders of magnitude higher (30 mV vs 0.45 mV). Simulations describe how the electromechanical interaction among fibers, which takes place at the microscale level, affects the polarization measured along the fiber length. To save computational time, simulations are performed on fibers shorter than those used in the device, after verifying that increasing the length of the fiber by a given factor leads to an increase of the output voltage by roughly the same factor (Figure 2e). An analysis of the behavior of the single fiber also highlights the dependence of the piezoresponse on the shape of the cross-section (i.e., circular vs rectangular), with a significantly improved performance by circular fibers (2.2 times under the same applied force, Figure 2f). Modeling the stacking of fibers with rectangular cross sections leads to the description of the bulk piezoelectric samples, which can be used as the basis of comparison. In the arrays, the interfiber interaction can be analyzed depending on the stacking directions of individual nanostructures. Elliptical cross sections are additionally studied, indicating an improved piezoelectrical response when the long axis of the ellipse is parallel to the direction of the applied force (Figure 2g). Building an array along the planar direction (Figure 2h) leads to a remarkable enhancement of the piezoresponse by increasing the number of adjacent cylindrical fibers. This mechanism takes place when the array starts to be built and it is mainly because of the electromechanical interaction among adjacent cylindrical fibers, which generate a *cooperative* effect in the plane of the array that restrains the transverse deformation and correspondingly increases transverse stresses. On the contrary, the voltage output does not increase upon placing many fibers with rectangular cross-section in mutual contact (Figure 2h). A more-complex cooperative effect is found upon building an array along the out-of-plane direction. In this case, both the reduction of the mechanical stiffness along the out-of-plane direction (because of the increased thickness) and the interfiber electromechanical contact interactions concur to an enhancement of the piezoresponse by up to 2 orders of magnitude with respect to a bulk film. The interfiber cooperative behavior corresponds to asymptotic effective piezoelectric coefficients of *d̅*_31_ = 19.6 pC/N and *d̅*_33_ = −29.3 pC/N. The picture that emerges from this analysis can be applied to other classes of nanofibers, regardless of their constituent material or fabrication process, and it stimulates further work in the field. This mechanism has been exploited in several device configurations to define energy harvesting devices and sensors.^40^ In addition, cooperativity of nanofibers enables (i) enhanced sensitivity to differently oriented mechanical forces and long-term durability in all-organic e-skin sensor^41^ and (ii) mimicking the spatiotemporal human perception, exhibiting a mechanosensitivity of ∼0.8 V kPa^–1^, in cross-linked networks of electrospun gelatin nanofibers with out-of-plane stacking.^42^

**Figure 3:**
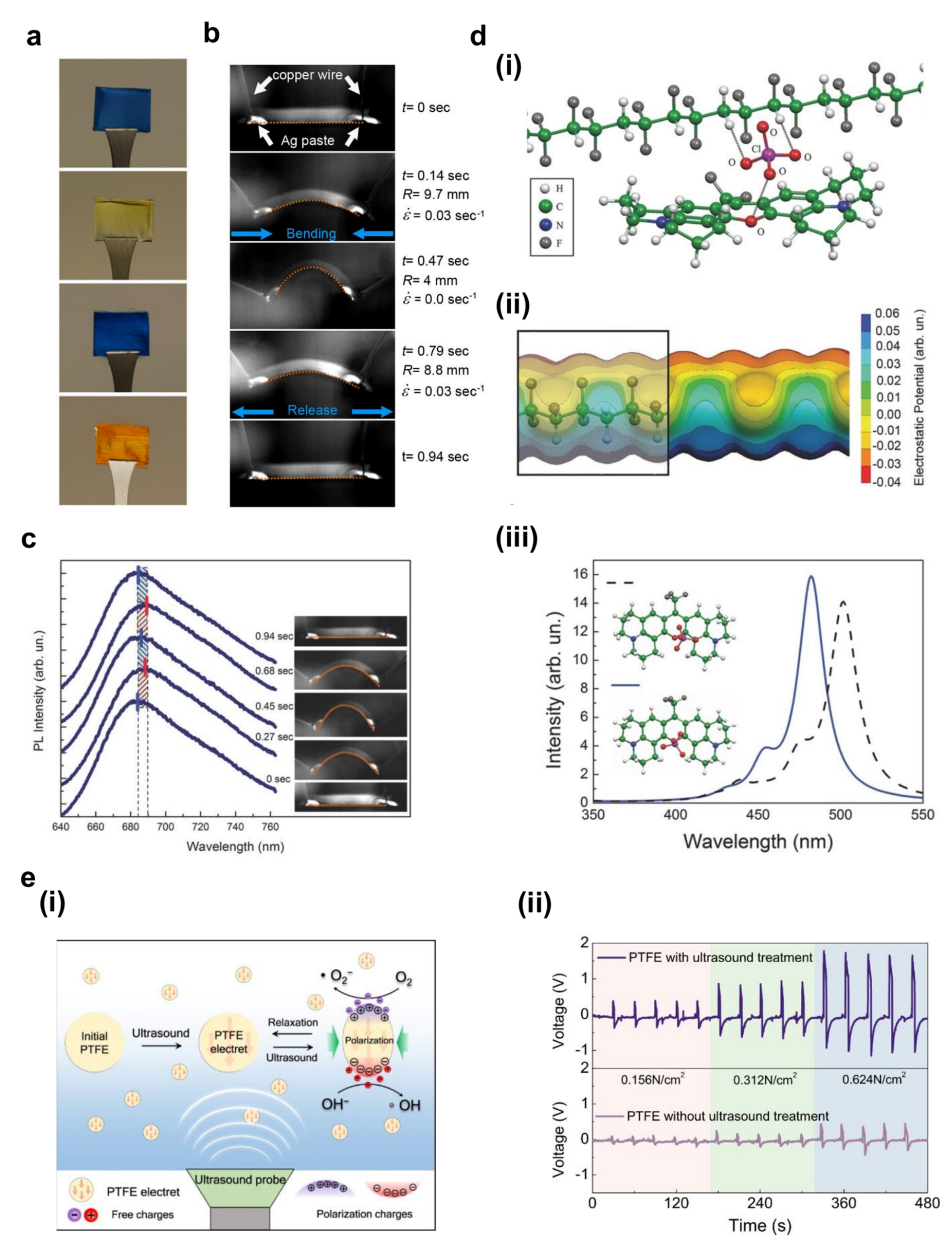
Piezoelectricity enabling tunable optical and chemical functionalities in hybrid systems. (a) Photographs of arrays of hybrid nanofibers. (b) Sequence of frames captured during bending of the hybrid nanofibers. *R* and ε̇ are the bending radius and strain rate at each time, respectively. (c) PL spectra at different times during bending and the corresponding photographs. (d) Results from DFT calculations capturing the electrostatic interactions among the counterion dye and the polymer chain (i), (ii), and (iii) Calculated DFT absorption spectra corresponding to the two different conformations of the system, reported in the inset. Reproduced with permission from ref (43). Copyright 2017 John Wiley and Sons, Inc. (e) (i) Scheme of the generation of reactive oxygen species by PTFE nanoparticles under ultrasound irradiation. (ii) Output voltage vs time of PTFE membranes, before (bottom) and after (top) activation with ultrasound waves, under normal forces. Reproduced from ref (45). Copyright 2021 The Authors. http://creativecommons.org/licenses/by/4.0/.

At the molecular level, cooperative coupling phenomena may unveil unusual mechanisms and open possibilities for applications in several fields. We found that electromechanical coupling through piezoelectric polymer chains of P(VDF-TrFE) plays a role in new hydrid materials exhibiting multifunctionality, such as concomitant piezoelectricity and light emission.^43^ It is possible to precisely control the optical properties through mechanical forces applied to flexible materials, in a reversible fashion. The test system used for these experiments consists of a bendable, flexible array of P(VDF-TrFE) nanofibers doped with a counterion dye exhibiting emission peak at about 700 nm. The dye is incorporated into the polymer solution prior to electrospinning, and both the emissive properties of the dye and the piezoelectric properties of the polymer are not individually affected by processing. To broaden the cases studied, different dyes and nonpiezoelectric polymers have been combined to form arrays of fibers. The final color of the realized arrays depends on the specific dye used (photographs in Figure 3a). The emission of the counterion dye exhibits a reversible red shift upon applying dynamic stress during bending experiments performed by using two synchronized linear stages. A high-speed camera records the bending movement and determines the instantaneous values of the curvature radius, *R*, and strain rate, ε̇ (Figure 3b). At the maximum amplitude of bending, *R* is as low as 4 mm, and the strain rate zeroes. Large values of a dye emission red shift are recorded at *R* ≈ 5 mm and ε̇ ≈ 0.04 during both the application and the release of the tensile stress. Such behavior is cyclic, and it can be captured by measuring photoluminescence (PL) during bending movements (Figure 3c). No significant shift is found in nonpiezoelectric polymers and nanowires without counterions. By first principle density functional theory (DFT) calculations performed by the Della Sala group,^43^ we could rationalize this behavior by evidencing that photophysical properties are correlated with mechanical stresses applied to electrostatically interacting molecular systems (*mechanophores*) in the nanofibers. The counterion interacts with the dye molecule and with the positively charged hydrogens of the P(VDF-TrFE) piezodipoles, thus forming a coupled system (Figure 3d(i) and (ii)). When a strain is applied, a spatial shift between the polymer chains and the dye is induced, thus driving the coupled system to different local potential energy surface states. Each of these configurations exhibits different optical properties in terms of absorption and emission wavelength. The maximum achievable theoretical shift of the main absorption peak is 20 nm (Figure 3d(iii)). These findings suggest that the electrostatic coupling with piezoelectric materials at the molecular level might enable additional functionalities in complex materials at the macroscale and make them capable of monitoring local mechanical stress by providing a measurable optical signal as output.

A different application of how piezoelectric materials can drive additional functionalities has been demonstrated in the field of organic synthesis. In mechanochemistry, the piezoelectric effect has been exploited by Kubota et al.^44^ to induce the selective formation of chemical bonds similarly to photoredox catalysis processes, through the formation of a piezoelectric potential in barium titanate (BTO) nanoparticles stimulated by ball milling. Under mechanical impact, polarized nanoparticles can transfer electrons to small organic molecules, thus activating redox-active components in organic synthesis. The so-proposed mechano-redox method has the potential to strongly reduce the environmental impact of various chemical processes, because it does not require the use of complex reactors, high quantities of organic solvents, or inert atmosphere. Another route to piezocatalysis exploits piezoelectricity in poly(tetrafluoroethylene) (PTFE) nanoparticles electrets to trigger the generation of strongly oxidizing species, which can rapidly degrade organic pollutants and sterilize drinking water.^45^ Wang et al. used ultrasound waves to induce a permanent polarization in inert PTFE particles, which then exhibit a voltage above 1 V upon an applied pressure of 0.624 N/cm^2^ (Figure 3e).

## 4 Nanofibers in Energy Harvesting Device Platforms

**Figure 4:**
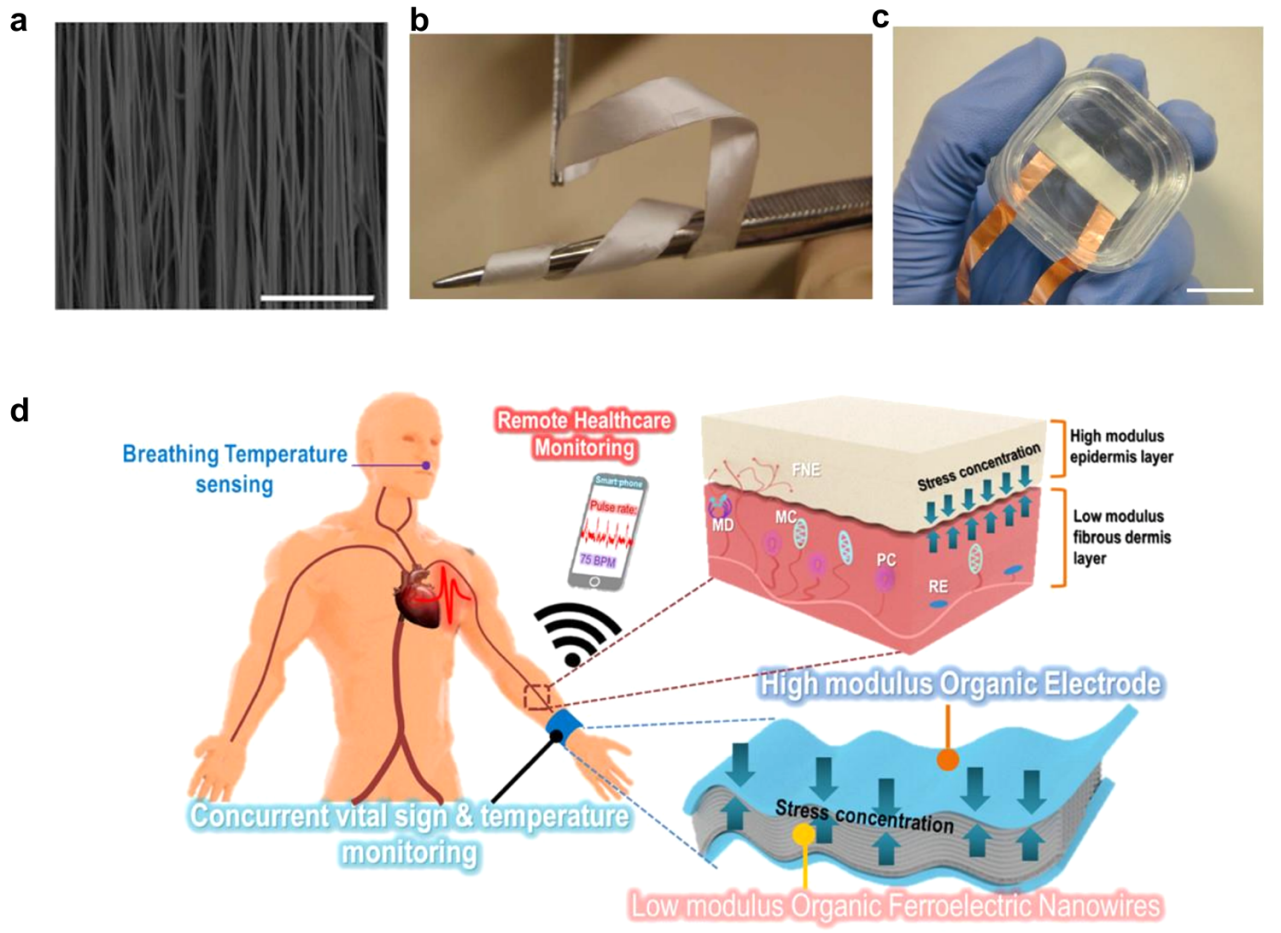
Aligned array of electrospun nanofibers for environmental sensing and physiological monitoring. (a) SEM image and (b) photograph of a dense array of aligned nanofibers. (c) Accelerometer and orientation sensor based on aligned nanofibers. Reproduced with permission from ref (40). Copyright 2013 Nature Publishing group. (d) Schematics of the operational principle of a healthcare monitoring system based on nanofibers array. Reproduced with permission from ref (46). Copyright 2021 American Chemical Society.

**Figure 5:**
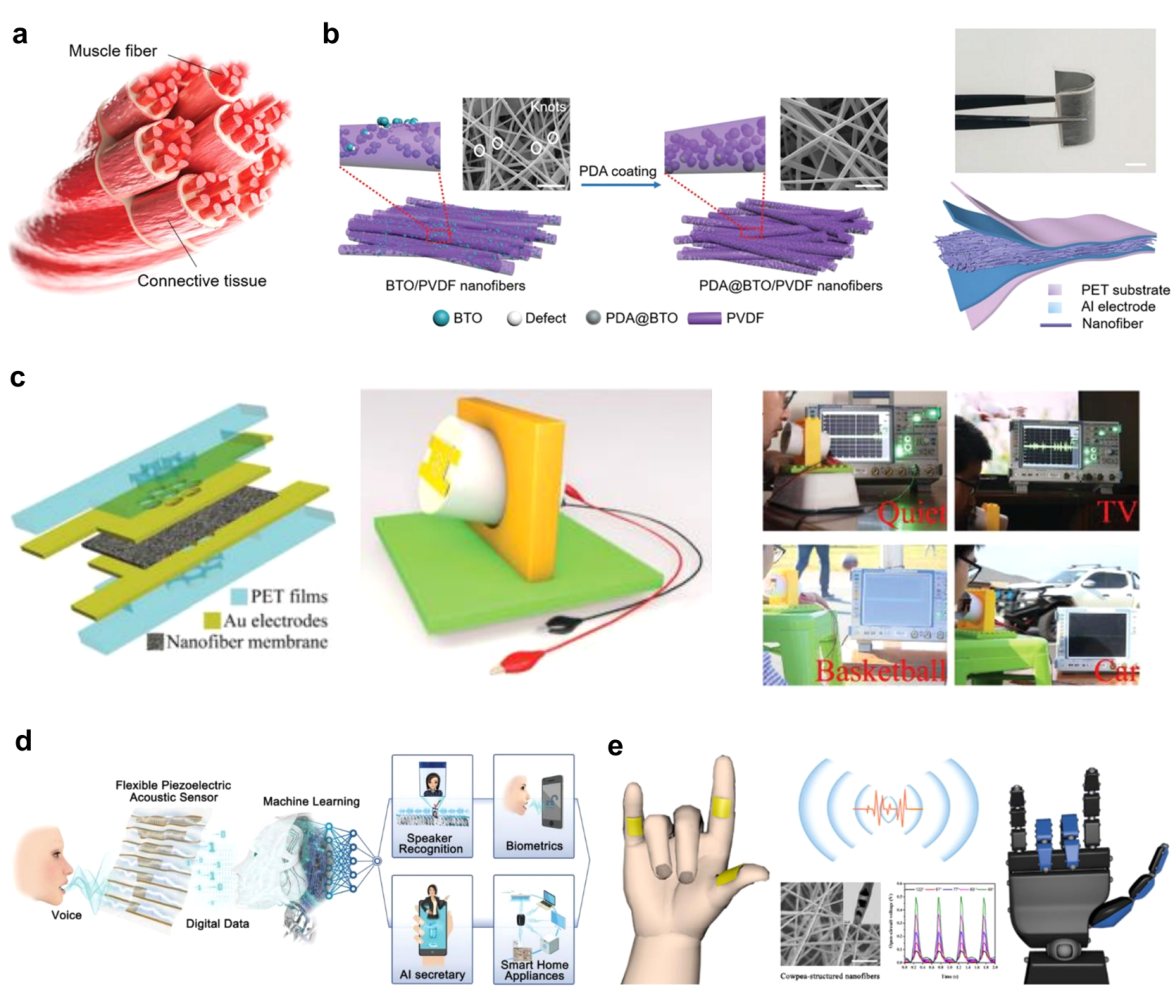
Highly sensitive nanofiber fabrication and its application toward artificial intelligence. (a) Scheme of the human leg connective tissue and muscle fibers. (b) PDA-coated BTO/PVDF nanofibers: fabrication strategy mimicking the human muscle fibers. Reproduced with permission from ref (48). Copyright 2021 John Wiley and Sons, Inc. (c) Schematic diagram of a voice recognition system based on PAN nanofibers and its operation in noisy indoor and outdoor environments. Reproduced with permission from ref (49). Copyright 2021 John Wiley and Sons, Inc. (d) Schematic diagram of voice user interface platforms where sounds can directly operate electronic systems. Reproduced with permission from ref (56). Copyright 2019 John Wiley and Sons, Inc. (e) Human-machine interactive wireless platform. Reproduced with permission from ref (50). Copyright 2019 Elsevier.

Electrospun piezoelectric nanofibers can be easily integrated within several different device platforms to build nanogenerators, environmental and physiological monitors, and voice recognition systems. Some representative examples are reported in Table 1.^40,46−55^ Highly dense arrays of PVDF-TrFE nanofibers (Figure 4a) are used as the pressure sensor, displaying ultrahigh sensitivity to detect pressures as small as 0.1 Pa.^40^ Three-dimensional, free-standing architectures of nanofibers (Figure 4b) show structural flexibility and large sensitive areas, and devices can be built simply by establishing electrical contacts at the ends of aligned fibers. The use of rotating collectors in electrospinning, together with high-boiling solvents for preparing the polymer solution to be electrospun, enable the formation of mesoscopic joints among adjacent fibers, which significantly enhances mechanical robustness and leads to superior piezoelectric properties. Vibration/acceleration and orientation sensors are demonstrated by using the array of nanofibers as a diaphragm across a hole opened in a plastic film, sealed over the closed cavity of a transparent box. About 10 μV peak-to-peak output voltage has been measured in response to environmental vibrations induced by sound pressure levels of 60–80 dB, in experiments carried out on our materials in the Rogers group (Figure 4c).^40^ These methods generated a vibrant field of research in the past decade. A similar strategy to align nanofibers can be used to build a piezoelectric harvester/nanogenerator based on all-organic components. Various architectures for health monitoring can be developed in this way. For instance, human physiological signals (pressure and temperature changes) can be successfully measured in real time and wirelessly transferred to a smartphone, evidencing that piezoelectric polymer nanofibers can be used in remote healthcare monitoring (Figure 4d).^46^ Here xylitol-mixed poly(3,4-ethylenedioxythiophene):poly(4-styrenesulfonate) films serve as top-bottom electrode and infrared heater and enable the simultaneous exploitation of mechanical (stretching) and thermal stimuli. An alternative approach to build effective infrared-sensitive heater was developed by the Xia group through the incorporation of Au nanocages in electrospun PVDF nanofibers.^47^ The hybrid fibers absorb the incoming light and convert it to heat and then to an electrical signal; in this way they can be useful to detect both tactile and near-infrared stimuli. The voltage output of these devices under localized pressure is found to be more than 1 order of magnitude larger than that of devices based on pristine polymer nanofibers, an effect that can be attributed to an enhancement of the piezoelectric β phase of PVDF because of the inclusion of the nanocages. Additional capabilities in wearable physiological monitoring are achieved by electrospinning composite nanofibers modified by polydopamine (PDA), in a device architecture inspired by the muscle-connective tissue (Figure 5a).^48^ The PDA improves the toughness of the nanofibers, and it leads to higher electromechanical coupling and hence improved piezoelectric response. The increased electromechanical coupling is associated with the enhancement of the interfacial adhesion between the filler and the polymer and therefore demonstrates a more effective stress transfer compared to pristine fibers (Figure 5b).^48^

**Table 1:** Nanofibers in Energy Harvesting Device Platforms

materials, fiber orientation	output response	applications	ref
PVDF-TrFE, aligned	1.5 V, 40 nA under bending test at 2 Hz	vibration/acceleration and orientation sensor	(40)
PVDF-polyaniline, aligned	45 V, 0.7 μA/cm^2^ under compressive force at 6 Hz	wearable pressure and temperature sensors	(46)
PVDF-Au nanocages, random	18.9 V, 27.4 nA under compressive force at 5 Hz	tactile sensor and NIR detector	(47)
PVDF/BaTiO_3_/ PDA, random	11 V, 0.4 μA under compressive force at 1 Hz	wearable pressure sensor	(48)
PAN, random	40.7 V at 115 dB	acoustic sensor	(49)
PVDF/ZnO, random	11 V, 550 nA under compressive force at 1 Hz	pressure and bending motion sensor	(50)
PVDF-TrFE, aligned	5 mV under tensile load	sensor of cardiac tissue contractions	(51)
PVDF-TrFE, random	21 V, 3.8 μA under compressive force at 1 Hz	power supply of electronic devices	(52)
PVDF, random	0.56 V, 15.58 nA under compressive force at 5 Hz	acoustic sensor	(53)
P(VDF-TrFE)/AlN, random	82 V under bending at 0.5 Hz	wearable sensors	(54)
PVDF/dopamine, random	16 V under compressive force at 1.2 Hz	pressure sensor	(55)

Airborne signal/noise is another abundant source of vibrations, which can be potentially harvested by the use of soft piezoelectric components, including nanofibers.^40^ To this aim, fibers can be sandwiched between two conductive electrodes, which are previously drilled to directly expose the active layer based on mats of polymer filaments to the sound pressure. For instance, a voice recognition system based on polyacrylonitrile (PAN) nanofibers is able to distinguish sounds coming from both musical instruments and people with high accuracy, even if positioned in noisy indoor or outdoor environments (Figure 5c).^49^ Based on this technology for acoustic sensing, it is possible to develop machine learning algorithms for speech processing, aiming at obtaining user-interface platforms where voice sounds can directly drive the operation of electronic systems (Figure 5d).^56^ Human–machine interactive platforms toward remote control of gestures are also being developed, on the basis of PVDF/Zinc oxide nanofibers sensitive to motion. These piezoelectric sensors show outstanding sensitivity for bending (4.4 mV/deg within the range from 44° to 122°) and fast response time (∼76 ms),^50^ whose performance allows for the realization of wireless robotic hands, synchronously performing the same gesture as human hands (Figure 5e).

## 5 Toward Sustainable Materials: Piezoelectric Nanofibers Based on Biodegradable Polymers

Since the discovery of piezoelectricity of wood and bone in the 1950s by Fukada et al.^57,58^ several efforts have been addressed to the investigation of piezoelectricity in biomaterials and ultimately to their use in energy harvesting devices. The use of piezoresponsive bioresorbable and biodegradable polymers represents an important milestone to build eco-friendly and more sustainable components and devices for electronics. A large variety of compounds, such as polysaccharides, polypeptides, and polynucleotides have been investigated in this respect. Although many biodegradable materials show poor mechanical robustness and fast degradation, some of them have been successfully processed and stabilized in the form of nanofibers and used in devices such as body-implantable transducers.^59^ In the following, some representative examples of piezoelectric electrospun nanofibers based on both natural and synthetic biodegradable polymers are reported (Table 2).^42,59−63^ These systems can constitute a highly interesting field of future research.

**Table 2:** Nanofibers Based on Biodegradable Polymers

material, fiber orientation	piezoelectric coefficient (pC/N)	output response	applications	ref
gelatin, random	*d*_33_ ∼ −20	1.8 V, 0.45 μA/cm^2^ under compressive force at 5 Hz	pressure sensor	(42)
PLLA, aligned	*d*_14_ ∼ 19	1.5 V under compressive force	pressure sensor and transducer	(59)
Silk, random	*d*_33_ ∼ 38	∼9 V under compressive force at 4 Hz	power supply of electronic devices	(60)
PLLA, aligned	*d*_33_ ∼ 27	∼700 mV under bending stress at 10 Hz	electrical stimulator of cells	(61)
PLLA/rGO aligned	−	5.5 V, 29 nA under bending	electrical stimulator of cells	(62)
PBLG, aligned in PDMS matrix	*d*_33_ ∼ 54	∼100 mV under bending	−	(63)

**Figure 6:**
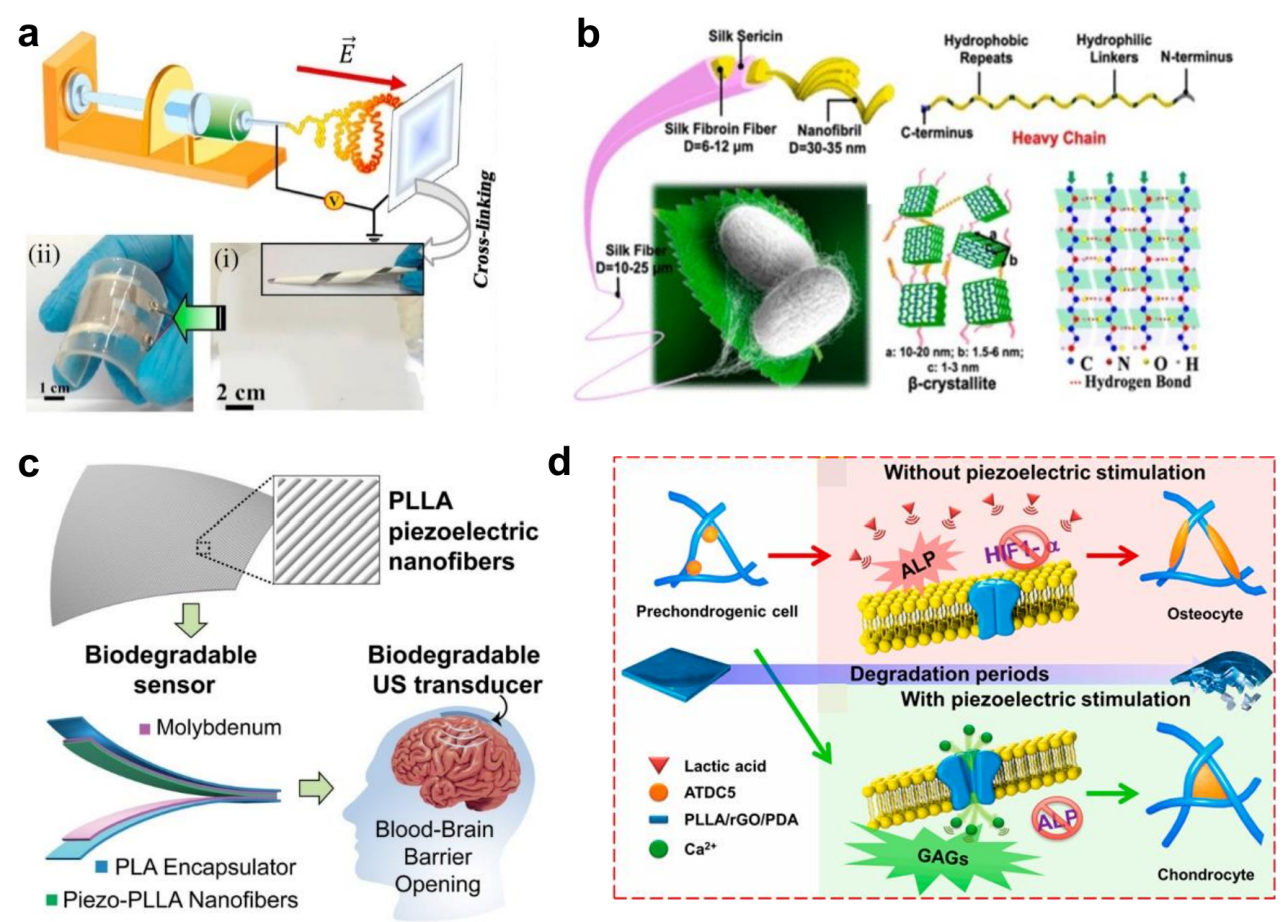
Piezoelectric nanofibers and devices based on biopolymers. (a) Fabrication scheme and photograph of mats and device based on gelatin nanofibers. Reproduced with the permission from ref (42). Copyright 2017 Elsevier. (b) Scheme of the cocoon silk fiber structure, hydrogen bonding network, and piezoelectric β-crystal form. Reproduced with the permission from ref (65). Copyright 2019 American Chemical Society. (c) PLLA nanofiber-based ultrasound transducer generating acoustic pressure for blood–brain barrier operation. Reproduced with the permission ref (59). Copyright 2019 National Academy of Science. (d) Mechanism of stem cell proliferation mediated by the concomitant effect of piezoelectric stimulation and scaffold degradation. Reproduced with the permission from ref (62). Copyright 2021 Elsevier.

Cellulose and chitin are the most abundant polysaccharides in nature, and both exhibit piezoelectric properties. Cellulose is the main constituent of plant cellular walls and of vegetable fibers (i.e., cotton), whereas chitin is a major component of cell walls in fungi and of the exoskeleton of arthropods. They are both fiber-forming polymers, with semicrystalline, ordered structure, and several known polymorphisms. In cellulose, hydroxyl groups arranged in a noncentrosymmetric order form a net dipole moment, which determines the piezoelectric properties of the material. Similarly, in chitin, an intrinsic molecular polarization is associated with the noncentrosymmetric crystal structure of both α- and β-polymorphs. These materials can be successfully electrospun, although they have rarely been incorporated into piezoelectric devices, partly because of their poor mechanical properties.^64^ Also gelatin, produced by partial hydrolysis of collagen extracted from the connective tissues of animals, exhibits piezoelectricity (with a *d*_33_ of −20 pC/N), and it can be reliably used within sensor platforms (Figure 6a), showing excellent operational stability (over 108,000 cycles) and antifatigue properties.^42^ Being derived from collagen, gelatin’s piezoelectric behavior origin has been associated with the dipole moment along the peptide chain axis and to the supramolecular interactions regulated by hydrogen bonding.^42^

Silk fibroin, derived either from *Bombyx mori* silkworm or other organisms, such as spiders, is composed of long amino acid sequences of repeating units of glycine, serine, and alanine (Gly-Ser-Gly-Ala-) (Figure 6b). It is a semicrystalline block copolymer. An antiparallel β-sheet architecture might be present, with a monoclinic unit cell.^65^ The application of elongational external forces results in a high degree of polymer chain orientation and in an improved piezoelectric response. In electrospun silk fibroin, the measured longitudinal piezoelectric coefficient (*d*_33_) is about 38 ± 2 pC/N,^60^ corresponding to a 20-fold improvement with respect to spider silk.

PLLA is a transparent, flexible plant-derived biodegradable polymer that may exhibit piezoelectricity because of the presence of C=O dipoles along the chain in a helical configuration. The thermodynamically stable phase of PLLA is the α-form, which features a random orientation of dipoles along the main chain, resulting in a zero net dipole moment and no piezoelectricity. The C=O dipoles of β-PLLA are instead aligned with respect to the backbone chain, thus exhibiting a nonzero total dipole moment. The application of an external shear stress, such as thermal stretching, enables the rotation of the C=O dipoles thus promoting their preferential alignment and the establishment of an electrical polarization. The elongational forces exerted during electrospinning also favor the formation of the β-crystalline form,^66^ and the resulting fibers show shear piezoelectricity (*d*_14_ ∼ 19 pC/N) superior to bulk films.^59^ It is also possible to modulate the piezoelectric response by changing the electrospinning parameters, for example, by reducing the final fiber diameter.^61^ An effective strategy to fabricate PLLA nanofibers showing stable, effective, and highly controllable piezoelectric performance has been found by the Nguyen group.^59^ An annealing procedure followed by cutting the fibers at 45° with respect to the fiber direction allows for increasing the degree of crystallinity and exploiting shear piezoelectricity by maximizing shear force under an applied normal pressure, thus leading to the fabrication of an ultrasonic transducer that can be implanted into the brain to open the blood–brain barrier, enable the delivery of drugs with a minimally invasive procedure, and ensure self-degradation of the used implant (Figure 6c).^59^ Scaffolds based on PLLA have additionally been used in dynamic modes, i.e., under external mechanical stimuli, to investigate the influence of piezoelectricity on stem cell differentiation.^61,62^ Results on prechondrogenic ATDC5 cells indicate that the combined effect of scaffold degradation and electrical stimulation favors the differentiation into either chondrocytes or osteocytes, depending on the stimulation intensity (Figure 6d). In addition, it is possible to enhance stem cells differentiation toward specific lineages by exploiting normal or shear piezoelectricity in the scaffolds. A normal voltage might enhance neuronal differentiation, whereas a shear voltage promotes the osteogenic differentiation.^61^

Poly(γ-benzyl, l-glutamate) (PBLG) is a synthetic polypeptide, achieving a rod-shaped, α-helical conformation when dissolved in organic solvents. Intramolecular hydrogen bonds formed between −NH and −CO groups confer stability to the helix-structure, and large dipole moments can be oriented by poling. Electrospinning promotes poling of the PBLG molecules. Although such fibers show high thermal stability and superior piezoelectric properties, they cannot sustain deformation for long time because cracks forming at the surface may accelerate degradation and water permeation.^63^ However, upon embedding such fibers within elastomeric matrices, it is possible to stabilize the mechanical and piezoelectrical performance, to build force sensors with sensitivity up to 615 mV N^–1^ and a maximum (peak-to-peak) voltage generation of 200 mV.^63^

## 6 Conclusions and Outlook

This Account highlights recent advances in piezoelectric polymer nanofibers made by electrospinning as functional material for sensors and energy harvesting systems. We focus on guidelines, derived from both experiments and modeling, for enhancing the piezoelectric properties of polymer nanofibers and exploiting the shear behavior in simple device geometries. Intercoupling effects at molecular up to interfiber length scale are investigated to unveil the piezoelectric behavior of electrospun fibers within arrays, the electromechanical interaction among fibers, which takes place at the microscale level, and the effects on the polarization measured along the fiber length. At the molecular level, electromechanical coupling through piezoelectric polymer chains plays instead a role in new hydrid materials exhibiting multifunctionality, such as concomitant piezoelectricity and light emission. As examples of integration into device platforms, environmental and physiological monitors, and voice recognition systems are especially relevant. Such devices have high potential for use in everyday life, although their implementation on a large scale would likely require new lines of production for materials and architectures. These should enable flexibility in electronic system assembly, as well as the capability to tailor the material properties for specific requirements by controlling the nanofiber size, their geometrical arrangement in complex arrays, and the on-demand exploitation of shear or/and normal piezoelectricity. The use of biodegradable polymers, both natural and synthetic, offers further opportunities for future research although several challenges remain for their operational stability and lifetime. Stabilization processes such as cross-linking by chemical, physical, and enzymatic methods combined with electrospinning might allow for an improvement of various properties, such as increase of the tensile strength, controlled modulation of water permeability and swelling behavior, and achievement of net dipole moment for stable piezoelectric operation.
